# Making an Invalid

**Published:** 1875-05

**Authors:** 


### MAKING AN INVALID.

ONCE there was a delicate little girl who was early taught lessons of industry and the virtue of early rising. The teaching she received was not confined to precept. Her father and mother and other members of the family afforded practical illustrations of all they taught. To rise with the lark in summer, and long before daylight in winter, was the constant practice of that family. The little daughter must not be an exception. What if she is frail and delicate, early rising is good for children, and the morning air will make her feel fresh and vigorous. And besides, the discipline of the household requires that all the members of the family shall be prompt at breakfast in order that the table shall not be kept standing, and the enterprises of the day thereby be delayed. Of course one little child, and that "only a girl," must not be allowed to derange the affairs of a well regulated household. Consequently she was always called betimes in the morning, and, struggling with sleep, striving against nature, unrefreshed, feeling as if she would "give the world" for another hour's rest, she was obliged to be present at the early breakfast. A light meal, forced into an unwilling stomach, is followed by a day of hard work for which the feeble body is poorly prepared. Six hours in school, two hours of practice at the piano, and, to avoid any danger of falling into habits of idleness, the remainder of the day occupied in some useful employment about the house—this was the usual routine of duties which this little girl was required to perform. And so, with a mind too active and too ambitious, the feeble body was driven to labors which would have been too arduous for a strong and healthy young woman. The result was that she was always "so tired;" she was always in a condition of physical exhaustion. She had not time to rest. She never had enough sleep. The food she ate was wholesome and palatable, but it was imperfectly digested and of course afforded inadequate nourishment. It could not have been otherwise, for the nervous force was constantly taxed to the uttermost with unceasing labor and study, and the stomach, deprived of its share of the vital energy, was unable to perform the duty of preparing the food for assimilation. Determination and *will* can sometimes drive a weak and trembling frame through tasks which had seemed impossible, but they cannot urge the stomach to perform any unusual works of digestion. The less thought given to the stomach the better. But in the case of the little girl, her training and the course of life which she was compelled to follow, produced their natural effect. She was made an invalid for life. An affection of the stomach was produced which for years has continued with scarcely any alleviation, and from which she will doubtless suffer to the end of life.

This case is a clear and striking illustration of the early-to-rise policy, the "busy bee" model, which was once, and is now by a few, regarded as the greatest of all virtues. An enlightened regard for the true welfare of children would prompt to a very different course. And first, a child should never be called up in the morning. When he has had enough sleep he will awake and get up fresh and bright, and ready for the business of the day. Sleep is as necessary as food; the most wholesome in quality and liberal in quantity of the latter, cannot compensate for a deficiency in the former. And then, the child should be allowed some rest during the day. If delicate, he might be encouraged to take a short nap in the afternoon. Do not banter him on being lazy, and a "sleepy-head." Many a spirited little boy or girl, rather than bear such jeers from a parent, will go through tasks of study or other miscalled "duty," even to a degree of physical exhaustion which, if it does not terminate in brain disease, is quite sure to leave some permanent disability.

Do not crowd a child with his studies. If a prize is offered as a reward for excellence in any special branch, do not urge him to compete for it. It is better, in many cases, to prevent his entering the list at all.

We are always glad to hear that a boy under fifteen years old is "rather backward in his studies." In such cases it is likely that he is quite *forward* in health, and strength, and growth. The great duty of the parent is to see that the first fifteen years of a child's life is devoted to physical, and not to mental, development. We do not mean that his schooling should be entirely neglected; but that study and books should be *secondary*, and that the means which afford bodily health and vigor should be regarded as of the first importance. And finally, it is the height of refined cruelty to oblige a sickly or delicate little girl to sit two hours every day "practicing" at the piano. Half an hour in the morning and another half hour in the afternoon is enough for a strong and robust child.

---

In hundreds of thousands of cases of broken-down health, a brisk walk of an hour in the open air after breakfast, and then a nap of an hour or more before the midday dinner, and quiet for the rest of the day, will effect a cure.

---

There is wisdom in the old couplet,

Early to bed and early to rise,
Makes a man heathly, wealthy, and wise.

